# Idiopathic Thrombocytopenic Purpura (ITP) Complicated by a Hemorrhagic Ovarian Cyst and Hemoperitoneum: A Case Report

**DOI:** 10.7759/cureus.64260

**Published:** 2024-07-10

**Authors:** Sakshi Karmore, Gaurav S Mude

**Affiliations:** 1 Clinical Research, Datta Meghe Institute of Higher Education and Research, Wardha, IND; 2 Pharmacology, Datta Meghe College of Pharmacy, Datta Meghe Institute of Higher Education and Research, Wardha, IND

**Keywords:** splenectomy, romiplostim, intravenous immunoglobulin (ivig), corticosteroids, primary immune thrombocytopenia (itp), autoimmune disorder

## Abstract

Idiopathic thrombocytopenic purpura (ITP) is characterized by a persistently low platelet count, which can lead to serious bleeding such as gastritis and hemorrhagic stroke. The formation of auto-antibodies in ITP leads to increased destruction of platelets and then hampers hematopoiesis. Corticosteroids and intravenous immunoglobulin are among the common treatments used for ITP, but they have significant side effects. This is a case report of a 27-year-old woman with ITP who was found to be anemic, thrombocytopenic, and had a ruptured ovarian cyst after the initial romiplostim therapy. The patient benefited from fluid resuscitation, blood transfusion, and corticosteroid therapy; then, the patient’s condition improved. This case highlights the complications associated with managing ITP, emphasizing the importance of personalizing therapy regimens through regular monitoring to improve the balance of benefits and risk, resulting in a comprehensive treatment for chronic patients suffering from ITP.

## Introduction

Idiopathic thrombocytopenic purpura (ITP) is a major condition with low blood platelet counts. The treatment becomes complicated because this disease progresses unpredictably. Some people with ITP experience severe bleeding, and this can cause such dangerous events as hemorrhagic strokes and gastrointestinal bleeding, among others [1,2]. Two processes occur in the pathophysiology of ITP, namely increased platelet destruction and decreased production [3]. Platelets are misidentified by the spleen as foreign substances, leading to their prompt destruction by autoantibodies that the organ produces erroneously. Besides, these autoantibodies may disrupt bone marrow giant cells known as megakaryocytes, which generate blood platelets, causing a further drop in the number of these cells [4]. The goal of ITP administration and management is now to balance the risks of bleeding with the side effects of the treatment. Corticosteroids and intravenous immunoglobulin are widely used as first-line therapies for rapidly increasing platelet counts. However, these treatments often lead to serious adverse effects such as weight gain, high blood pressure, and infection, which negatively affect patients’ lifestyles [5]. Various other options exist for those who fail to respond to the primary therapy, like thyroid peroxidase receptor agonists and splenectomy, though these have their own challenges [6]. This calls for constant monitoring of ITP management due to the risk of recurrence, and hence individualized treatment options are needed [7]. Doctors therefore need to strike a balance between managing the outcomes of the disease, perhaps through long-term immunosuppression, and looking at their patients’ psychological well-being regarding the chronicity of this disease [8].

## Case presentation

A 27-year-old woman suffered from ITP for a long time, and the first treatment started with romiplostim. There were dizziness, severe stomach pain, and many ecchymoses on the patient's body. Itchy rashes began three days prior to falling ill, which was followed by sudden abdominal pain that started around the patient's belly button twenty-four hours ago and was accompanied by feelings of generalized exhaustion and throwing up. Tachycardia and hypotension were detected (pulse rate 110/min, blood pressure 80/50 mmHg). The physical examination revealed epigastric discomfort and petechiae on her chest, abdomen, and extremities. Investigations confirmed severe anemia (Hb 7.3 g/dL), profound thrombocytopenia (platelet count 4,000/µL), and hemoperitoneum with a left-sided hemorrhagic ovarian cyst on ultrasound. Management included fluid resuscitation, blood and platelet transfusions, and methylprednisolone therapy. Surgical and OBGY consultations recommended conservative management over ovarian artery embolization, resulting in the administration of leuprolide and a total of 16 units of RDPs and two units of whole blood during hospitalization. The patient improved hemodynamically and was discharged on a regimen of weekly romiplostim, continued corticosteroids, and planned follow-up for hematological and gynecological evaluations.

Investigations

Initial workup included complete blood count showing Hb 7.3 gm/dL, platelets 4,000/µL, WBC 20,300/µL, and peripheral smear with microcytic hypochromic anemia. Ultrasound of the abdomen revealed a hemoperitoneum with a significant amount of free fluid and a left-sided hemorrhagic ovarian cyst. β-hCG levels were 2.39 mIU/mL, excluding pregnancy. Serial CBCs post-transfusion indicated rising Hb levels and platelet counts with stabilization of hematologic parameters.

Treatment

**Figure 1:**
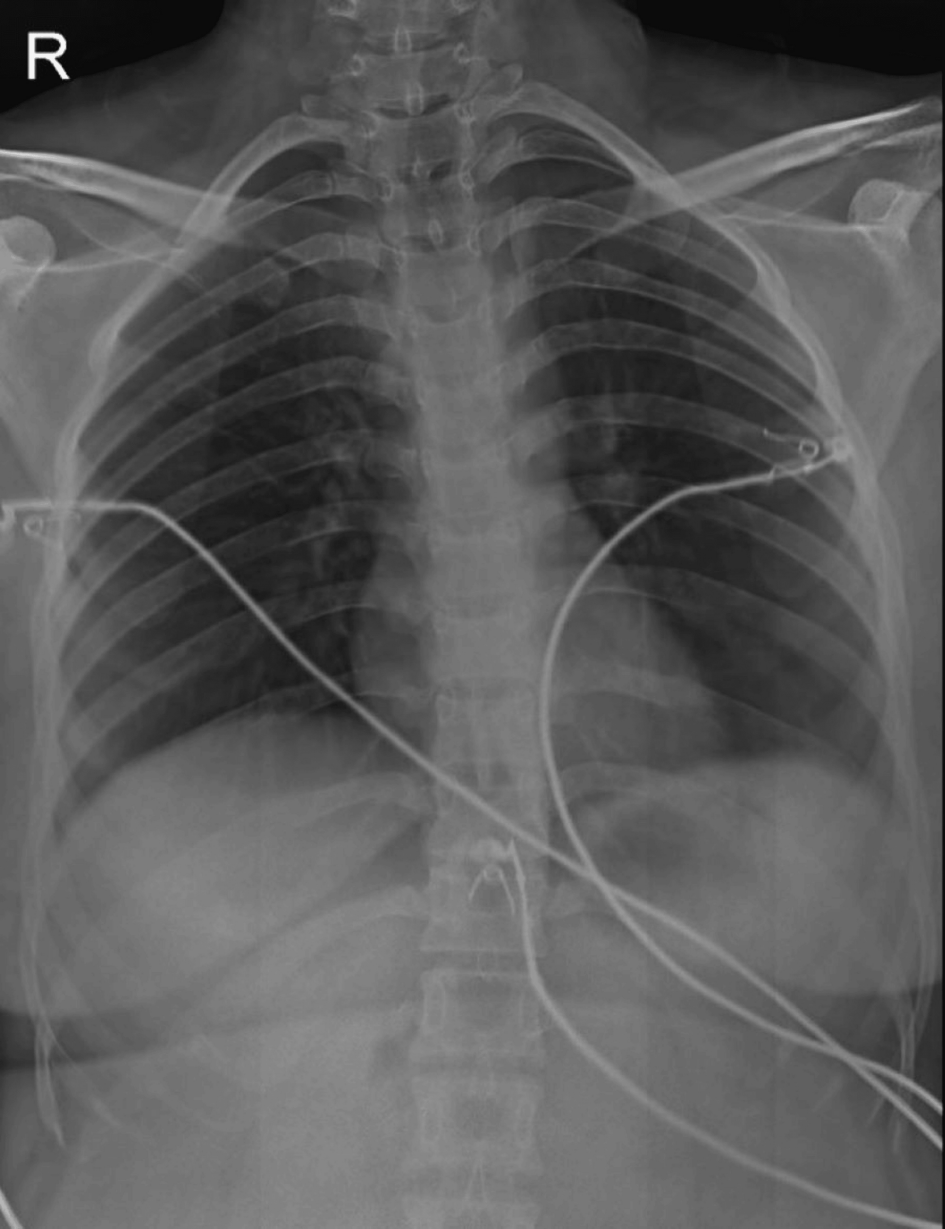
Chest radiograph of the patient on presentation

The treatment started with the patient receiving urgent fluid resuscitation with normal saline and Ringer lactate. Hemodynamic support included whole blood and platelet transfusions. The patient was administered to boost platelets and reduce inflammation including romiplostim (250 mcg IV stat), methylprednisolone (1 gm IV for one day, followed by 500 mg IV for two days), and adjunctive therapy with leuprolide (3.75 mg IM every three months). Antibiotics (metronidazole and levofloxacin), proton pump inhibitors, antiemetics, and analgesics were provided for symptomatic relief. The patient was discharged with medication to take at home including amlodipine (to control high blood pressure), Ferium XT (iron supplement to treat anemia), zebineuron (vitamin supplement), and tramadol hydrochloride/acetaminophen, with instructions for a weekly check-up with the doctor has been done to monitor hematological and gynecological issues. The chest X-ray demonstrated normal lung fields and bony structures, with a correctly placed NG tube and CVC. No acute abnormalities were noted (Figure 1).

After an X-ray, a laboratory examination was done. The patient’s blood test results indicated anemia with a hemoglobin (HB) level of 7.3 g/dL and an RBC count of 3.03 million cells/mm^3^. MCV (74.3 fL) and MCH (24.2 pg/cell) suggested microcytic hypochromic anemia. An elevated WBC count (12,900 cells/mm^3^) indicated potential infection or inflammation, while a low platelet count (0.1 × 10⁵/mm^3^) (Table 1) and potassium level (2.9 mEq/L) (Table 2) suggested thrombocytopenia and hypokalemia, respectively.

**Table 1:** Complete blood count investigations with a cell counter.

Lab variables	Results	Reference range
Hemoglobin %	7.3	12–16
Mean Corpuscular Hemoglobin Concentration (g/dL)	32.6	31–35
Mean Corpuscular Volume (fL)	74.3	76–100
Mean Cell Hemoglobin (pg/cell)	24.2	25–32
Total RBC Count (million cells/mm 3)	3.03	0–1.070
Total WBC Count	12900	3,500–9,000
Total Platelets Count (× 10^5^ )	0.1	1.5–4.5
Hematocrit (%)	22.5	3–7
Monocytes (%)	02	3–7
Granulocytes (× 10^ 9^)	77	1.5–8.5
Lymphocytes (%)	20	25–45
Eosinophils (%)	01	1–3
Basophils (%)	00	0–0.75
Red Cell Distribution Width (%)	20.2	11.5–14.5

**Table 2:** Kidney function test and other tests β-hCG  = Beta human chorionic gonadotropin

Lab variables	Results	Reference range
Kidney Function Test		
Urea (mg/dL)	27	6–24
Creatinine (mg/dL)	0.6	0.5–1.5
Sodium (Na+) Serum (mEq/L)	141	135–145
Potassium (K+) Serum (mEq/L)	2.9	3.5–5.0
Other Tests		
β-hCG	2.39	

**Figure 2:**
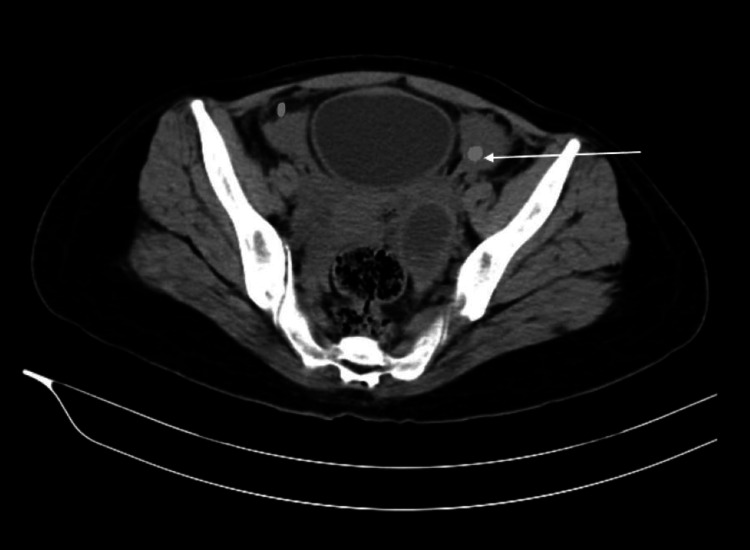
Contrast-enhanced computed tomography of the abdomen and pelvis of the patient at presentation. In the left ovary: a hemorrhagic cyst measuring 4.3 x 3.5 x 5.1 cm was found

After an X-ray and laboratory examination, a further radiological examination with contrast-enhanced computed tomography (CECT) in the abdomen and pelvis was done (Figure 2). However, a substantial anomaly was discovered in the left ovary: a hemorrhagic cyst measuring 4.3 x 3.5 x 5.1 cm was found. This cyst was distinguished by a peripherally enhancing cystic lesion with a hyperdense non-enhancing component, indicating internal bleeding. The scan also detected significant hyperdense fluid in the belly and pelvis, which suggests hemoperitoneum, as well as mild bilateral pleural effusion. Clinical correlation was recommended to further appreciate the implications of these findings.

## Discussion

This 27-year-old woman with a long history of ITP presented in a manner that represents the complexity of the above-mentioned hematologic disorder when combined with an acute abdomen condition. The symptoms mentioned by the patient were being dizzy, severe pain in the abdomen, a general body rash, tiredness, feeling sick, and vomiting which show a very serious progression of the disease, ITP, which is an autoimmune condition leading to platelet damage with chance of bleeding [7]. However, tachycardia or rapid heartbeat together with hypotension or low blood pressure meant that this patient had hemodynamic instability; therefore, this was a clear indication that she was suffering from ITP which does not deteriorate because of hemorrhage. Laboratory results showed thrombocytopenia (platelet count 4000/µL) and profound anemia Hb (7.3 g/dl) which required immediate intervention. The increased level of WBCs (20,300/µL) indicated the possible occurrence of an acute phase reaction to bleeding or infection. Peripheral smear showing microcytic hypochromic anemia is suggestive of chronic blood loss or hemolysis which is characteristic of severe ITP exacerbations [9]. Imaging studies with the use of ultrasound and CECT confirmed the patient’s symptoms as acute abdomen due to hemoperitoneum and a hemorrhagic left ovarian cyst contributing to the patient’s hemodynamic instability [10]. The patient's condition was stabilized by the fluid resuscitation administered and transfusion of blood products. This acute anemia and thrombocytopenia were managed through crystalloids, whole blood, and platelet transfusion while managing hemorrhagic complications of ITP [8]. Methylprednisolone reduced auto-immune platelet destruction while romiplostim, a thrombopoietin receptor agonist, was still used to stimulate platelet production as per ITP treatment protocols [11]. Due to the patient's overall health and the associated risks, surgery was deemed to be the best course of action for this patient rather than intrusive methods such as ovarian artery embolization or conservative treatment with leuprolide for a hemorrhagic ovarian cyst [12]. The patient's blood contained many leucocytes; therefore, the patient had to take antibiotics including metronidazole and levofloxacin which are potent enough to avoid the chances of infection. They also used other drugs for patient support which were antiemetic, analgesic, or proton pump inhibitors in order to alleviate some of the symptoms that the patient manifested [13]. Depending on the discharge plan, there is outpatient monitoring with ongoing romiplostim, steroid, and hematological as well as gynecological assessment appointments. This approach includes the ITP outpatient follow-up. It is possible to avoid its repetition with terrible consequences. Amlodipine is an anti-hypertensive drug, Ferium XT is an iron supplement, zebineuron is a vitamin supplement and tramadol hydrochloride/acetaminophen is a pain killer [5]. The above instance is a clear case of problems faced in ITP management like spontaneous bleeds and gynecologic complications. The following excerpt describes how these issues may be addressed using a team-based approach. The immediate stabilization of the patient without any delay, correct drug dosing, and safety are important [6,8].

## Conclusions

A woman suffering from ITP for a long time had complained about stomach aches, and there were misunderstandings that proved the existence of an incurable form of the disease. Bruises and vital findings showing extreme weight loss, as well as low blood counts leading to anemia and thrombocytopenia, required immediate medical attention. The imaging showed intraperitoneal bleeding and hemoperitoneum, which made the situation more serious. The patient was stabilized with fluids, given a blood transfusion, and received platelet transfusions after ending with steroids. Managing ITP conservatively is done in combination with monitoring the ovarian cyst using romiplostim while availing outpatient services as well as full recovery based on a team approach by different specialists. 
